# Diagnostic Role of Multi-Detector Computed Tomography in Acute Mesenteric Ischemia

**DOI:** 10.3390/diagnostics14121214

**Published:** 2024-06-07

**Authors:** Francesco Michele Ronza, Teresa Letizia Di Gennaro, Gianfranco Buzzo, Luciana Piccolo, Marina Della Noce, Giovanni Giordano, Giuseppe Posillico, Luigi Pietrobono, Francesco Giuseppe Mazzei, Paolo Ricci, Salvatore Masala, Mariano Scaglione, Stefania Tamburrini

**Affiliations:** 1 Department of Diagnostic Imaging, AORN “S. Anna e S. Sebastiano”, 81100 Caserta, Italy; 2 Intensive Care Unit, AORN “S. Anna e S. Sebastiano”, 81100 Caserta, Italy; 3 Radiology I, Fondazion e IRCCS Policlinico San Matteo, 27100 Pavia, Italy; 4 Unit of Diagnostic Imaging, Azienda Ospedaliera Universitaria Senese, University of Siena, 53100 Siena, Italy; 5 Unit of Emergency Radiology, Department of Radiological, Oncology and Patological Sciences, “Sapienza” University of Rome, 00185 Rome, Italy; 6 Department of Medicine, Surgery and Pharmacy, University of Sassari, 07100 Sassari, Italy; 7 Department of Radiology, Ospedale del Mare-ASL NA1 Centro, 80147 Naples, Italy

**Keywords:** acute mesenteric ischemia, MDCT, atherosclerosis, embolism, dissection, NOMI, mesenteric thrombosis

## Abstract

Mesenteric ischemia diagnosis is challenging, with an overall mortality of up to 50% of cases despite advances in treatment. The main problem that affects the outcome is delayed diagnosis because of non-specific clinical presentation. Multi-Detector CT Angiography (MDCTA) is the first-line investigation for the suspected diagnosis of vascular abdominal pathologies and the diagnostic test of choice in suspected mesenteric bowel ischemia. MDCTA can accurately detect the presence of arterial and venous thrombosis, determine the extent and the gastrointestinal tract involved, and provide detailed information determining the subtype and the stage progression of the diseases, helping clinicians and surgeons with appropriate management. CT (Computed Tomography) can differentiate forms that are still susceptible to pharmacological or interventional treatment (NOM = non-operative management) from advanced disease with transmural necrosis in which a surgical approach is required. Knowledge of CT imaging patterns and corresponding vascular pathways is mandatory in emergency settings to reach a prompt and accurate diagnosis. The aims of this paper are 1. to provide technical information about the optimal CTA (CT Angiography) protocol; 2. to explain the CTA arterial and venous supply to the gastrointestinal tract and the relevant ischemic pattern; and 3. to describe vascular, bowel, and extraintestinal CT findings for the diagnosis of acute mesenteric ischemia.

## 1. Introduction

Intestinal ischemia is a sudden decline in blood flow through the mesenteric vessels [1] and, based on symptoms, it can be stratified into acute, chronic, and acute-on-chronic forms [2].

Acute intestinal ischemia is a rare abdominal emergency that accounts for 1–2% of acute gastrointestinal disease [3,4,5], although it carries a high mortality rate due to the non-specific presentation and rapid progression of the disease [1]. Mortality has been reported in up to 90% of cases if the diagnosis is delayed and transmural bowel wall necrosis occurs [6,7]. Acute intestinal ischemia may be caused by arterial or venous occlusion, nonocclusive disease, and bowel strangulation [8].

Clinical presentation and laboratory tests are usually nonspecific [9]. Patients complain of acute abdominal pain out of proportion to otherwise benign findings on physical examination [2]. Laboratory parameters, such as l-lactate, leukocytosis, and D-dimer, are often elevated only in advanced disease, suggesting the development of an acute abdominal surgical disease [10]. New intestinal biomarkers have been proposed such as ischemia-modified albumin (IMA), intestinal fatty acid-binding protein (I-FABP), D lactate, and L citrulin, although their use in clinical practice is still not diffuse [10,11,12].

Imaging is necessary for acute mesenteric ischemia (AMI) diagnosis; however, there are significant differences in sensitivity among imaging techniques, with multiphasic CT being considered the best diagnostic tool. Plain X-ray can demonstrate a gasless abdomen in early ischemic injury, followed by bowel loop dilatation, but these findings are not specific for AMI. Late findings are also intraperitoneal free gas and portal vein or parietal pneumatosis, suggesting a necrotic/perforative evolution. Mesenteric vessels can be evaluated by ultrasound, particularly using the Doppler technique, but with low sensitivity [13]. Ultrasound can also detect dilated and hypoperistaltic bowel loops, but this finding is not specific for AMI, so ultrasound is sometimes an initial work-up technique to exclude other causes of abdominal pain [14]. Magnetic Resonance Imaging is an effective technique in AMI diagnosis [15], but its feasibility in an emergency scenario is low, while angiography is now considered a therapeutic technique more than a diagnostic one. Laparotomy is considered a gold standard, but it is often too late [10].

Therefore, a sudden onset of acute abdominal pain and the need for morphine are considered suggestive of AMI. In these patients, a prompt multiphasic CT should be performed [16].

Contrast-enhanced Multi-Detector CT Angiography (MDCTA) is the imaging of choice for the diagnosis of intestinal ischemia. MDCTA can detect the vascular occlusion of mesenteric arteries or veins and demonstrate downstream bowel wall injury. Moreover, MDCTA allows differential diagnosis between intestinal ischemia subtypes, which is mandatory to guide the correct management [17]. Therapeutic management requires a multidisciplinary approach of general or abdominal surgeons, vascular surgeons, interventional radiologists, and intensivists, according to the recommendations of WSES (World Society of Emergency Surgery) [10]. In 2016, the first Intestinal Stroke Center (Structure d’urgences vasculaires intestinales [SURVI]) opened in France [16,18,19], improving the survival of patients who underwent multidisciplinary and combined treatment based on the presence of reversible or irreversible lesions. The identification of the subtype of intestinal ischemia is mandatory to achieve the most appropriate management. In intestinal ischemia, the bowel involvement is characterized by a three-stage disease progression. In stage I, the disease is reversible and pathologically characterized by necrosis, erosion, ulceration, edema, and hemorrhage localized to the mucosa. In stage II, necrosis extends into the submucosal and muscularis propria layers. In stage III, transmural necrosis involves all three layers [20,21]. In this scenario, MDCTA findings are fundamental for discriminating reversible ischemia from irreversible transmural necrosis [22]. While arterial occlusion patterns (atherosclerotic, embolic, and superior mesenteric artery dissection) can take advantage of endovascular treatment, venous and nonocclusive ischemia require medical treatment in early phases. Surgery, resections without anastomosis (damage control surgery), or revascularization are recommended when transmural necrosis and/or peritonitis occur to remove necrotic bowel loops and to avoid life-threatening systemic complications such as sepsis, disseminated intravascular coagulation, and multi-organ failure [10,19,23]. The prognostic role of MDCT in AMI has been recently proposed based on the skeletal muscle index (SMI) [24], calculated by dividing the skeletal muscle areas by the height squared, supporting the prognostic capabilities of CT that could help discriminate between survivors and non-survivors. SMI could be an adjunctive parameter to serum lactate level, hemoglobin level, C-reactive protein level, and white blood in predicting patient outcome [25,26].

MDCTA diagnosis requires a specific CTA protocol, knowledge of arterial and venous bowel supply and the relevant ischemic pattern, and confidence with diagnostic pitfalls that may be encountered. The accurate analysis of CT findings supports clinicians, interventional radiologists, and surgeons in staging the disease in reversible and irreversible forms.

## 2. MDCTA Technical Consideration

MDCTA is the best imaging modality in the diagnosis of acute mesenteric ischemia, with a sensitivity of 94% and a specificity of 95% [9,21,27,28]. The optimal CT protocol includes a multiphasic examination: non-enhanced acquisition followed by biphasic contrast-enhanced phases (with a delay of 30 s for the arterial phase and at 60–70 s for the venous phase). Intravenous nonionic iodinated contrast material at a high flow injection rate (4–5 mL/s), followed by a saline solution flush (1.5 mL/kg), is administered optimizing scanning times with contrast bolus tracking methods [21,29]. The delayed scan phase, at 3 min from contrast injection, can be considered in a case-by-base evaluation to detect delayed or decreased enhancement. Multiplanar Reconstruction (MPR) and Maximum Intensity Projection (MIP) images are extremely useful in identifying vessel thrombosis and reduced bowel wall enhancement, reason why the optimal thickness of image acquisition is 1–2 mm [30]. Oral contrast is not indicated because it obscures the bowel wall that should be accurately examined to assess hypoperfusion.

Non-enhanced CT images are useful for detecting spontaneous bowel wall hyperdensity and/or intraluminal content hyperdensity that are suggestive of bowel ischemia [31,32], and to assess atherosclerotic calcifications [33]. Biphasic contrast-enhanced CT allows the easy detection of arterial and venous filling defects and bowel wall enhancement. The role of Dual-Energy CT (DECT) is still controversial [34,35,36]. Virtual non-enhanced images can be derived from enhanced scans, although radiologists should be confident to correctly identify a hyperdense bowel wall and intraluminal content.

The identification of bowel ischemia can be better identified in the conventional 120 kVp-like images [34,35]. Instead, a diagnosis based only on the iodine map can negatively influence sensitivity [35]: iodine quantification on an iodine map distribution should be added to the primary evaluation of CT images to increase diagnostic confidence [35,37,38] because the real role of iodine mapping using a semi-quantitative color scale in the diagnosis of bowel ischemia is still controversial, and artifacts such as pseudo hyperenhancement caused by air-filled bowel segments, peristalsis related artifacts, and streak artifacts from metal should be taken into to account [34,35,36].

## 3. Intestinal Vascular Supply

Three main branches, originating directly from the abdominal aorta, supply the gastrointestinal tract: the celiac trunk, the superior mesenteric artery (SMA), and the inferior mesenteric artery (IMA).

**Figure 1:**
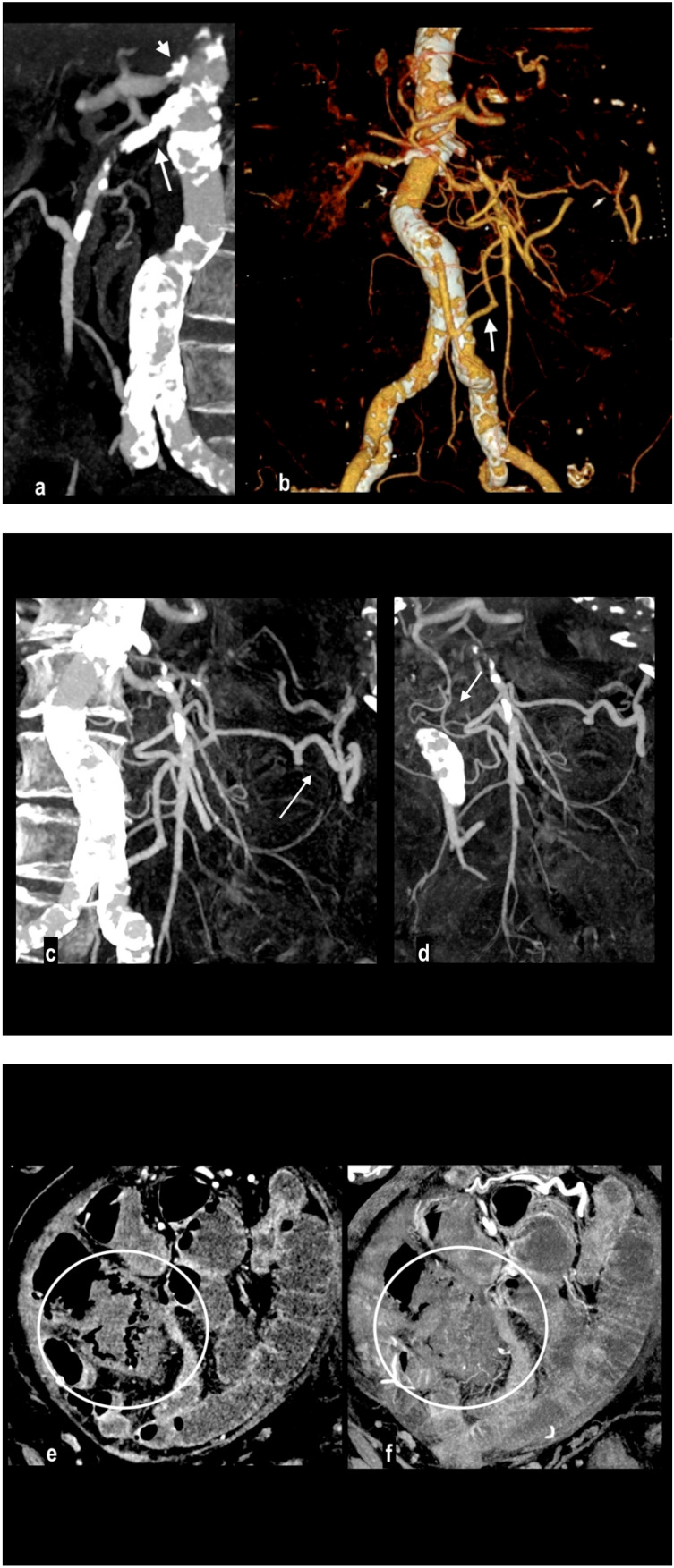
Abdominal pain in a 91-year-old woman with diffuse calcified vascular atheromasia. MIP arterial reconstruction (**a**) showed calcified occlusion of the proximal SMA (arrow) and critical ostial occlusion of the celiac trunk (arrowhead). VR reconstruction (**b**) well depicted an ectasis mesenteric inferior artery and a dilated Riolan artery (arrow), that allowed the collateralization of the middle SMA and its branches. MIP reconstructions showed a large caliber of splenic flexure arteries ((**c**) arrow) and pancreatico-duodenal arteries ((**d**) arrow). However, a watershed territory (ileocecal junction) did not receive appropriate hematic flow and caecum necrosis developed, with hypoenhancing wall thickness and parietal pneumatosis (circle; (**e**) coronal MPR; (**f**) coronal MIP).

All of them have a rich net of collateral branches: the pancreaticoduodenal artery between the celiac trunk and SMA, through the common hepatic artery; the Riolan arc and Drummond marginal artery between SMA and IMA. Watershed territories among these three districts are at higher risk of ischemia, particularly in hypoafflux conditions (Figure 1): the ileocecal junction, the splenic flexure (Griffith’s point), and the rectosigmoid junction (Sudeck’s point) [39].

SMA is the dominant artery involved in acute intestinal ischemia. SMA is anatomically divided into three segments (proximal, middle, and distal) based on the origin of the inferior pancreaticoduodenal artery and ileocolic artery. This classification should be taken into account in CT reports because the site of stenosis/occlusion and corresponding ischemic territories are fundamental in determining therapeutic choices [40].

Intestinal venous return is based on superior and inferior mesenteric veins, the first draining directly into the portal vein, and the second draining into the splenic vein. The mesenteric–portal system presents multiple collateral communications with the systemic venous circulation.

## 4. Mesenteric Ischemia CT Patterns

Mesenteric ischemia CT patterns can be related to vessel, bowel, and extraintestinal findings and are strictly related to the onset time of ischemia (Table 1).

### 4.1. CT Vessel Findings

There are two main categories of arterial acute intestinal ischemia: occlusive and nonocclusive [9]. Vascular findings precede bowel wall alterations, and the occlusive form includes the embolization (40–50%) or thrombosis (25–30%) of the superior mesenteric artery (AMS) and mesenteric venous thrombosis (10–18%) [10].

#### 4.1.1. Acute Arterial Mesenteric Ischemia

Arterial intestinal ischemia is a consequence of the sudden reduction of arterial blood supply that overcomes mesenteric vascularization reserves. It is usually determined by the critical stenosis or occlusion of SMA. Ethologically, embolic disease accounts for up to 50% of cases [2,41]; in the remaining patients, atherosclerosis, SMA dissection, and vasculitis are observed.

**Figure 2:**
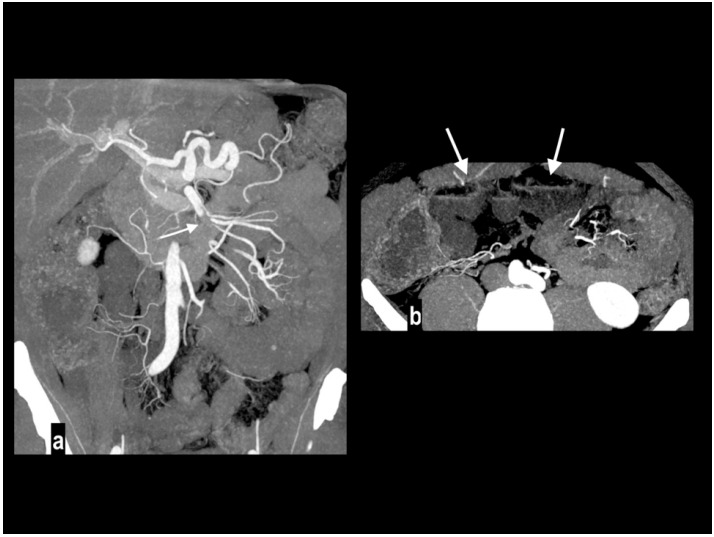
Embolic mesenteric ischemia in a 57-year-old man with atrial fibrillation. Coronal MIP arterial image (**a**) showed embolic occlusion of middle SMA (arrow), with preserved jejunal arteries enhancement and right colonic vessels collateralization. Hypoenhancing wall was appreciated only in distal ileus ((**b**) arrows).

Embolic disease is usually a manifestation of underlying cardiovascular disease [42] (atrial fibrillation, endocarditis) or, less frequently, aortic or mesenteric plaques, and it usually involves a high-flow SMA due to the narrow take-off angle from the aorta [2]. The topography and extent of the involved segment depend on the location of the embolus (Figure 2): the inflow to the proximal jejunum is preserved if the embolus is located near the takeoff of the middle colic artery, sparing inferior pancreaticoduodenal branches, or almost complete ischemia of the small intestine occurs if the embolus is located close to the SMA orifice [2,39,43,44].

**Figure 3:**
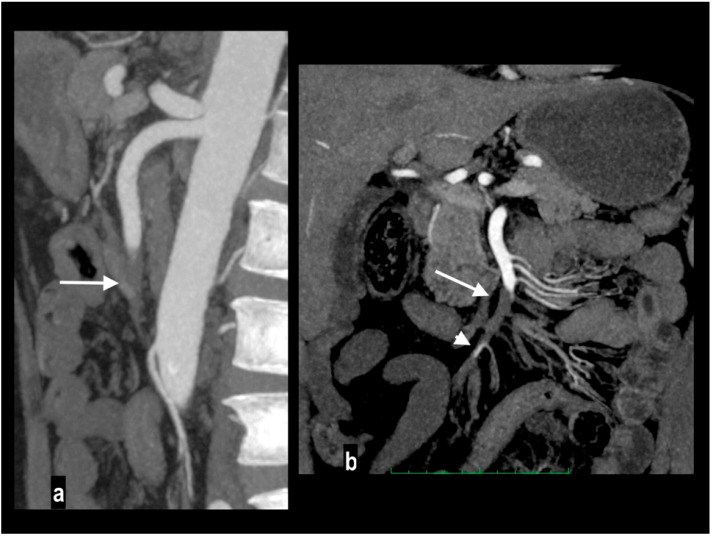

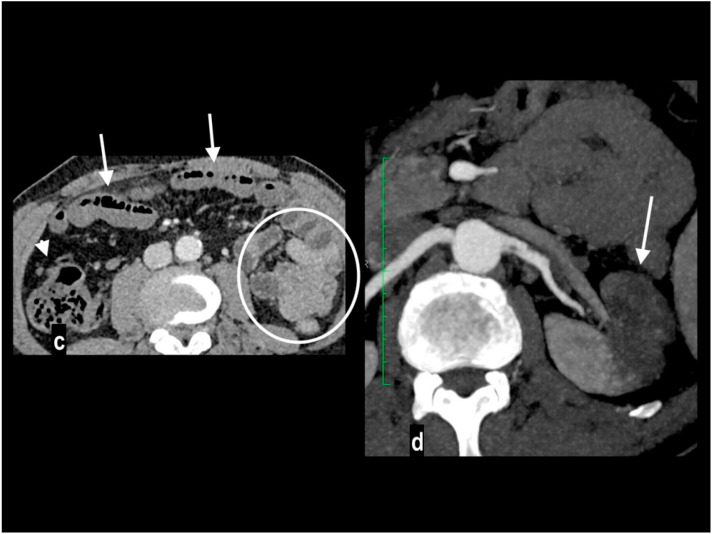
Acute abdominal pain in a 52-year-old with atrial fibrillation. Oblique sagittal and coronal MIP arterial images showed embolic occlusion of the middle SMA (arrow), with subtle peripheral segmentary enhancement of distal lumen (arrowhead) by a collateral vessel (**a**,**b**). Hypoenhancing ileal (arrow) and right colon (arrowheads) walls were well appreciated in comparison to jejunal loops (circle) on left abdominal side (**c**). A left renal infarct (arrow) was also present (**d**). Patient underwent a right colectomy.

Involved vascular beds are usually healthy and show poor collateralization, so clinical presentation and evolution to transmural necrosis occur earlier; moreover, concurrent emboli can involve other splanchnic arteries, particularly renal and splenic ones, determining parenchymal infarcts (Figure 3).

**Figure 4:**
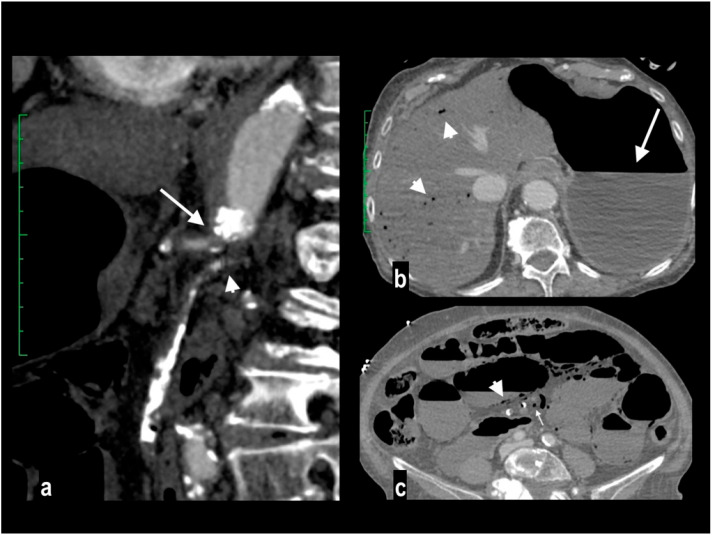
A 78-year-old woman with massive mesenteric ischemia. Atherosclerotic occlusion of the origin of the celiac trunk (arrow) and the proximal SMA (arrowhead) (**a**). Intrahepatic pneumatosis (arrowheads) and gastrectasia (arrow) (**b**). Hypoenhancing dilated paper-thin bowel wall with parietal pneumatosis (arrowheads) and mesenteric venous pneumatosis (arrow) were reported (**c**). The patient died a few hours after emergency room access.

Atherosclerotic steno-occlusion is usually observed in older patients with a history of systemic vasculopathy, and the risk of AMI increases when a critical stenosis involves two of the three major visceral arteries (Figure 4).

Intestinal ischemia in atherosclerotic disease is frequently associated with a >90% stenosis of SMA or a >70% stenosis of both the celiac artery and SMA [45]. Because of the long-time progression of atherosclerotic disease, an extensive network of arterial collaterals is recruited. When complete thrombosis occurs, it usually involves the ostia, determining ischemia of a long bowel segment [44]. Chronic mesenteric ischemia symptoms (postprandial pain, weight loss, etc.) can be present in patients’ history before the development of acute ischemia (acute-on-chronic form). At CT, the intra-arterial thromboembolic material can appear hyperdense on unenhanced CT, and high-density erythrocyte-rich thrombi (>36 UH) have been reported in transmural intestinal necrosis [46]. After contrast IV administration, an abrupt interruption of luminal enhancement is seen in vessel occlusion. The SMV/SMA (SMV, superior mesenteric vein) diameter ratio is measured at the level of the upper part of the kidney; a ratio ≥ 1 is considered normal, indicating the absence of a smaller SMV sign, while a ratio < 1 is indicative of acute SMA occlusion [47,48]. The sensitivity and specificity of the so-called “smaller SMV sign” for the detection of acute SMA occlusion have been reported at 70% and 99.2%, respectively, and it has been assessed in non-contrast images, advocating its utility in case of unsurpassable contraindications to the use of contrast agents [47].

**Figure 5:**
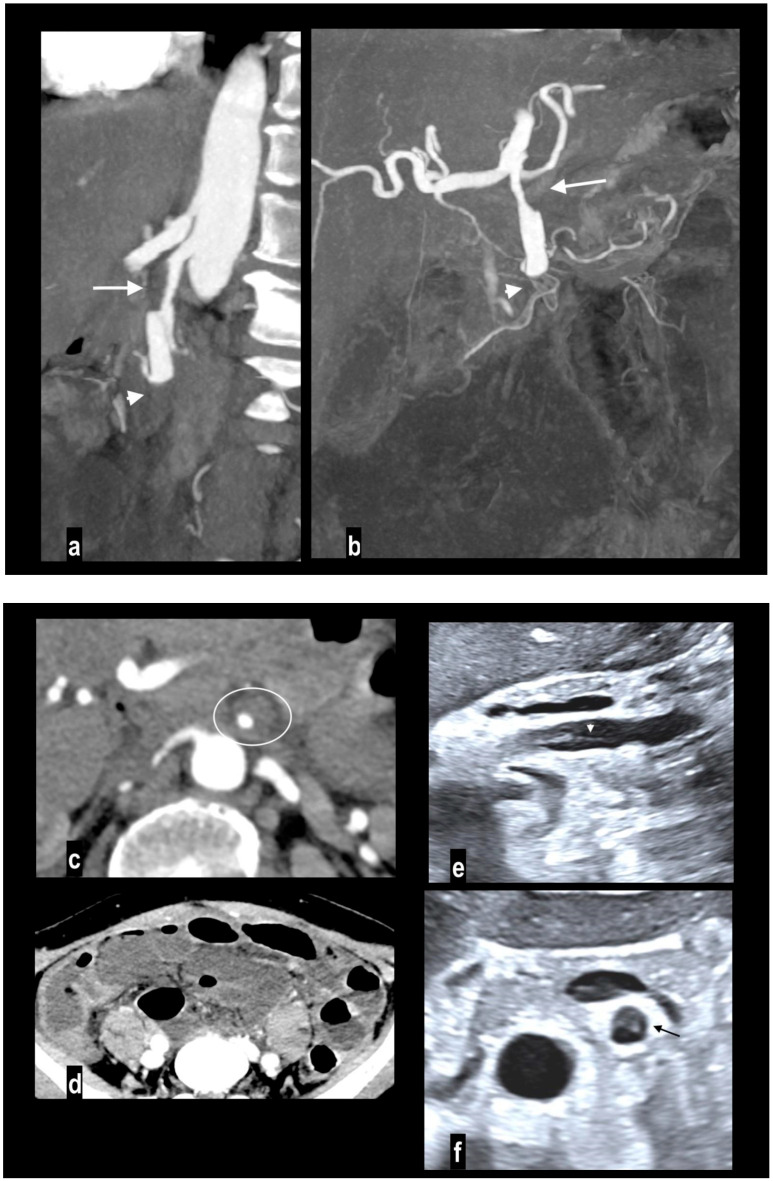
SMA dissection in a 42-year-old woman. MIP arterial images (**a**,**b**) showed segmentary false lumen thrombosis in the proximal convex side of SMA, with lumen narrowing (arrow). The vessel caliber had increased ((**c**) oval). Distally, the intimal flap continued in a complete vessel thrombosis (arrowhead), determining bowel ischemia with hypoenhancing paper-thin bowel walls (**d**). Proximal false lumen thrombosis (arrow) and intimal flap (arrowhead) were also well depicted at ultrasound examination performed before MDCT (**e**,**f**). The patient underwent surgical resection of necrotic loops.

An uncommon cause of arterial thrombosis is SMA dissection, which can occur as a continuation of aortic dissection or in isolation [49]; spontaneous isolated superior mesenteric artery dissection is defined as superior mesenteric artery (SMA) dissection without the presence of aortic dissection [50]. SMA spontaneous dissection is a rare cause of mesenteric ischemia (<5%) in males between the fourth and the fifth decade with no particular medical history [17], and typically affects the convex surface of the SMA trunk, at a distance of 1 to 3 cm away from the root [51]. Many classifications have been proposed for SMA dissection [50,52,53,54] to determine the shape, location, and extent of the false lumen, and whether the false lumen is thrombosed or the true lumen is stenotic. SMA dissection is responsible for acute pain and may or may not determine intestinal ischemia. Mesenteric ischemia in SMA dissection occurs when critical luminal stenosis or occlusion by false lumen thrombosis determines vessel occlusion and consequent bowel involvement [50]. The CT findings of SMA dissection are focal dilation, intimal flap, intramural hematoma, false lumen thrombosis, increased fat attenuation around the SMA, and mesenteric hematoma (Figure 5) [55,56]. Rarely, large vessels mesenteric arteritis can develop mesenteric ischemia as a devastating complication when parietal inflammation produces critical luminal stenosis or occlusion, presenting acutely and often requiring resection [57].

#### 4.1.2. Nonocclusive Mesenteric Ischemia

**Figure 6:**
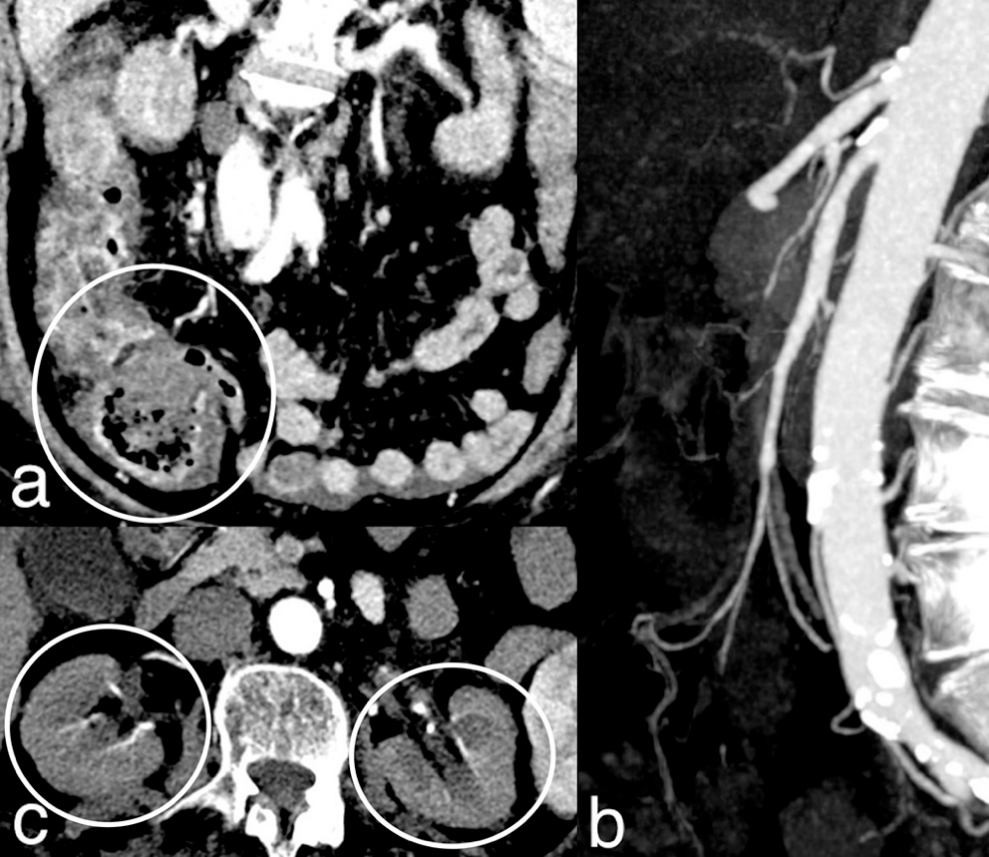
NOMI. Diffuse stratified wall thickening of right colon ((**a**) circle) with patent mesenteric arteries (**b**). The 78-year-old patient had a severe systemic hypoperfusion and multi-organ failure, with bilateral kidney cortical necrosis ((**c**) circles) and ARDS. The patient died a few hours later.

Nonocclusive mesenteric ischemia (NOMI) is an intestinal hypoperfusion in the absence of arterial or venous thromboembolism [2] accounting for up to 20% of cases of AMI [9,58], and atherosclerosis is not considered a risk factor [59]. The real incidence may be underestimated because this condition occurs in critically ill patients with predisposing conditions such as heart failure, major trauma, the use of vasopressors, and cardiogenic or septic shock (Figure 6) [2,42,60]. NOMI should be considered in the differential diagnosis in these patients because imaging findings may be subtle, and the distribution of the affected bowel may be discontinuous or involve multiple vascular territories [60]. The pathogenetic mechanism is poorly understood. It may rely on a protective reflex in which the mesenteric vessels undergo constriction or spasm to preserve blood flow to the cardiac or central nervous systems [2,3,21,42,61]. At CT, the diagnosis of NOMI can be challenging because vessels are not occluded and may present only a subtle luminal narrowing of SMA and its first-order branches. Segmental focal narrowing and dilatation (the so-called “string-of-sausages” sign) can be appreciated along mesenteric vessels. Although both the large and small intestine can be involved, characteristically, the bowel alterations are discontinuous and segmental. Other abdominal signs of the CT hypoperfusion complex can be appreciated such as a small-caliber aorta, a collapsed inferior vena cava, bowel mural hyperenhancement, and a hyperenhancement of the kidneys and adrenal glands [2,6,11,17,19,60]. Parenchymal infarcts are often associated with NOMI.

#### 4.1.3. Veno-Occlusive Acute Mesenteric Ischemia

Mesenteric ischemia by venous occlusion is determined by porto-mesenteric vein thrombosis. It is the least common cause of mesenteric ischemia (5–20% of cases) and occurs in younger patients. Hypercoagulability syndromes (antiphospholipid antibody syndrome, protein C and S deficiency, etc.) or states (pregnancy or oral contraceptives); a decreased porto-mesenteric blood flow, particularly in chronic liver disease; and a porto-mesenteric involvement in neoplastic, necrotizing pancreatitis, inflammatory or traumatic concurrent pathologies are the more common risk factors [62]. Porto-mesenteric thrombosis may also occur for extrinsic compression from tumor encasement [60]. Primary mesenteric venous thrombosis without an underlying disease has also been described [63]. The clinical onset of venous thrombosis is subacute because ischemia develops gradually and more slowly than arterial ischemia. Venous bowel infarct requires the extensive involvement of the upstream peripheral arcade of the vasa recta branches. For these reasons, patients complain of subacute long-standing symptoms for about 2–4 weeks and may present nausea and vomiting [6].

**Figure 7:**
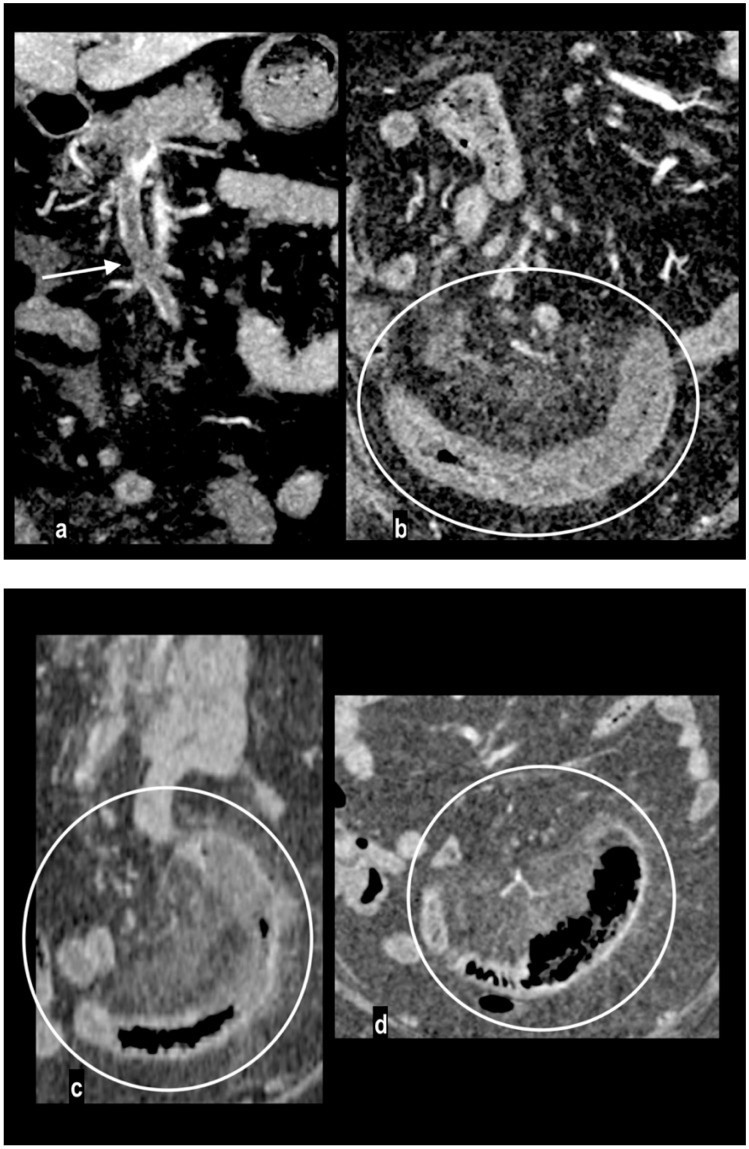
Abdominal pain in a 67-year-old female with a recent SARS-CoV-2 infection. Porto-mesentic partial thrombosis at coronal portal MPR ((**a**) arrow). Segmentary ischemic injury of an ileal loop with parietal hypoenhancing thickening and mild mesenteric haziness ((**b**) circle). The patient was treated with anticoagulant therapy. MDCT performed 4 and 7 days later ((**c**,**d**) circles) showed progressive parietal thinning with disappearance of intestinal wall line on mesenteric side, suggesting transmural necrosis. The patient underwent surgical resection of the necrotic loop.

Venous mesenteric thrombi are visualized as filling defects within the venous lumen. The vessel wall can appear as an enhancing rim. The venous outflow is interrupted, determining the engorgement of mesenteric veins [29]. Mortality in veno-occlusive mesenteric ischemia reaches 44% but is lower than in patients with arterial ischemia. Because intestinal necrosis occurs later due to the underlying pathomechanism, the first therapeutical approach is with anti-coagulation therapy that may improve the clinical picture in 24–48 h. When bowel necrosis occurs, only the damaged bowel loops are resected (Figure 7) [9,58,64].

### 4.2. Bowel Findings

**Figure 8:**
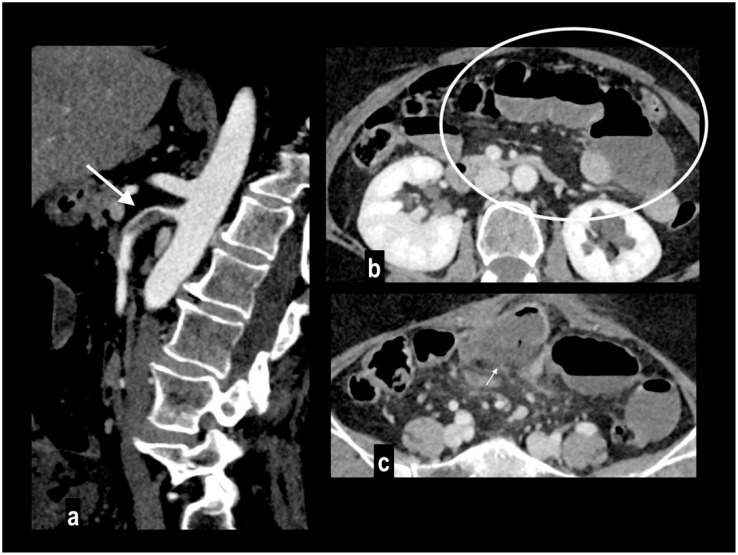
Acute abdominal pain in a 66-year-old woman. Atheromasic low-density plaque at the proximal SMA with luminal stenosis ((**a**) arrow). Dilated paper-thin small intestine wall ((**b**) circle). Segmentary transmural necrosis was observed on the mesenteric side of an intestinal loop (arrow), intestinal wall line loss and periparietal fluid effusion (**c**). The patient underwent surgical resection of the necrotic loop.

Immediately after acute arterial obstruction, the bowel shows a spastic reflexus ileus (gasless abdomen in plain X-ray), followed by hypotonia (hypotonic reflexus ileus) and parietal thinning (paper-thin sign) [38]. At CT, the bowel wall appears thinned (paper-thin) with decreased or absent parietal enhancement [2,49]. These findings are extremely specific for the diagnosis (up to 97–99%) [30,33], but they may be not evident in the early stage of the disease in which an accurate analysis of vessels should be carried out (Figure 8).

The comparison of parietal enhancement between an affected and a normal bowel in the arterial phase, particularly in segmentary ischemia, is extremely useful for the diagnosis [17].

**Figure 9:**
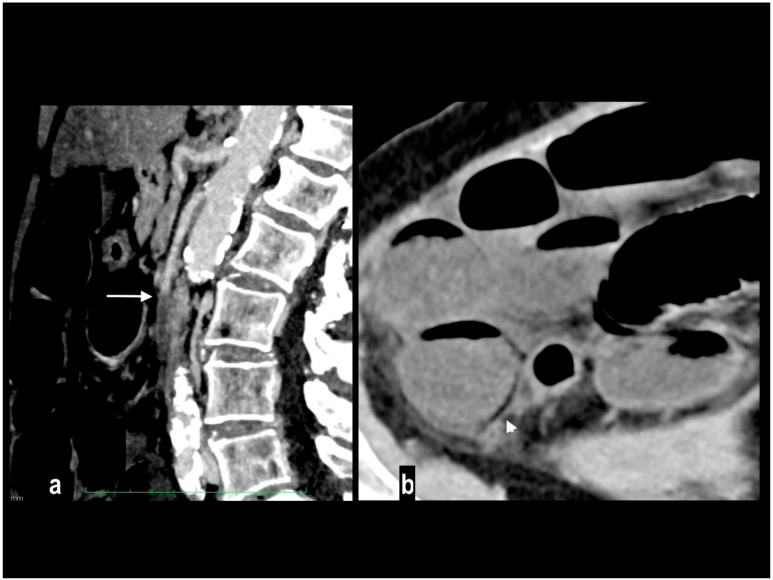
A 86-year-old patient presenting at the emergency department in coma. Blood lactate value was 14 mmol/L. An abdominal portal-phase scan showed SMA embolic occlusion ((**a**) arrow) and diffuse dilation of small intestine loops with segmentary parietal pneumatosis ((**b**) arrowhead). Multiple necrotic small intestine loops were found during surgery. The patient died on the following day.

Bowel wall pneumatosis has been reported in only 5% of patients with necrotic bowel at surgery [65] in advanced mesenteric ischemia, particularly when associated with porto-mesenteric pneumatosis (Figure 9). Pneumatosis and porto-mesenteric venous gas are the consequence of transmural necrosis with a translocation of luminal gas across the mucosa [2,49]. However, bowel pneumatosis is not a specific sign because it has also been described in patients with viable bowel, where it was a generic sign of mucosal integrity loss [66,67,68].

Reperfusion phenomena can be observed peripherally to bowel segments affected by arterial ischemia, due to collateralization, or when a partial or complete resolution of arterial obstruction occurs. Reperfusion is characterized by a re-established bowel blood flow after an ischemic injury, with oxidative stress and local inflammation, leading to bowel wall thickening with submucosal edema and mucosal and serosal hyperenhancement. This bull’s-eye appearance is similar to venous ischemic bowel wall changes, and, similarly, fat stranding and peritoneal fluid can also be observed [17]. Reperfusion phenomena are not necessarily associated with patients’ worsening and can also occur after a successful revascularization [20,69].

On the contrary, in veno-occlusive ischemia, the bowel walls are thickened with mural stratification due to acute congestion and edema, particularly evident in the submucosal layer [4,20]. Mesenteric haziness and stranding and peritoneal fluid are commonly detected due to vascular engorgement; there is impaired venous outflow with the extravasation of fluid into the mesentery [2,49].

At non-enhanced CT images, spontaneous bowel wall hyperdensity is observed as a consequence of intramural hemorrhagic infarction, while in a late phase, decreased enhancement and bowel wall thinning at enhanced phases are indicative of transmural necrosis [8].

These findings are also observed in NOMI, which is characterized by an association of arterial ischemia and reperfusion phenomena. Because of the segmentary bowel involvement of both the small and large intestines, a careful examination should be carried out to identify the segment involved that may correspond to watershed areas between vascular territories [60].

### 4.3. Extraintestinal Findings

In patients with mesenteric ischemia by embolic causes and in NOMI, it is mandatory to evaluate solid abdominal viscera. Systemic embolism or systemic hypoperfusion can produce wedge-shaped infarction in abdominal viscera, particularly in the liver, spleen, and kidneys [60], with prognostic implications. In the advanced phase, multi-organ failure can develop, with organ hypoperfusion and adrenal and renal hyperenhancement, determining the so-called CT hypoperfusion complex [70].

## 5. Conclusions

Acute mesenteric ischemia is a life-threatening condition that requires prompt diagnosis and treatment. MDCTA is mandatory in patients suspected of having mesenteric ischemia because it can confirm the diagnosis and allow a differential diagnosis between different types of intestinal ischemia. MDCTA also depicts several vascular, intestinal, and abdominal signs with prognostic and therapeutic implications. Radiologists should be aware of vessel, bowel, and extraintestinal findings to reach a definitive diagnosis, and knowledge of bowel vascular supply and CT findings is mandatory, especially in the early stage of the disease in which bowel findings may not be evident.

## Figures and Tables

**Table 1:** Main types of acute mesenteric ischemia and related CT findings.

	Atherosclerotic AMI	Embolic AMI	NOMI	Veno-Occlusive Mesenteric Ischemia
Cause	Atherosclerotic plaques at mesenteric artery origin	Embolism in distal SMA	SMA vasospasm	Mesenteric venous thrombosis
Early findings	Paper-thin hypoenhancing bowel wall (thickened with reperfusion)	Paper-thin hypoenhancing bowel wall (thickened with reperfusion)	Paper-thin hypoenhancing or thickened bowel wall	Thickened bowel wall with increased attenuation of mesenteric fat and peritoneal fluid
Late findings	Ill-marginated bowel wall with periparietal fluid effusion; bowel wall pneumatosis; porto-mesenteric/hepatic pneumatosis; free intraperitoneal air; extraintestinal findings (splenic and kidney infarcts; CT hypoperfusion complex)			

## Data Availability

Data are available on request.
